# Report of a Case of Monstrosity, with a Surgical Operation

**Published:** 1857-02

**Authors:** E. C. Moyer

**Affiliations:** Talbotton, Ga.


VOL. II.] FEBRUARY, 1857. [NO. 6.

### ORIGINAL COMMUNICATIONS.

### ARTICLE I.

*Report of a remarkable case of Monstrosity, with a Surgical operation.* By E. C. MOYER, M. D.

TALBOTTON, GA., Dec. 15th, 1856.

J. P. LOGAN, M. D.,

*Dear Sir :*—In compliance with a promise made you last summer, I report you the following case of a Monstrous Fœtus. On the 20th day of March 1842, I was called to see a negro girl belonging to Mr. Stephen Harvey, two years of age. She, together with several of the servants of the family, had been suffering much from inflammatory fever of a continued form. The child's abdomen from birth had been large and prominent. Shortly after the attack of fever, her abdomen rapidly enlarged, and about the 20th day, an opening was formed about an half inch to the right of the umbilicus, which afforded an exit (to use the language of master and mistress) to about one peck of most offensive matter, containing large locks of hair. At the time I saw her, she was laboring under hectic fever—pulse 140—profuse night sweats and diarrhœa, and great emaciation. Upon a very careful investigation of the case, I decided that the abdomen contained the imperfect rudiments of a Fœtus, and that nothing but its removal by an operation could save the life of the child, and so expressed myself to the master, assuring him at the same time, in all probability, she would die from the operation, in consequence of her great debility and emaciation ; but that the operation afforded the only ground upon which to predicate a hope of her recovery. On the evening of the 23d, three days after, I performed the operation, assisted by Dr's H. P. Smead, Thos. B. Turner and James Y. Gardner, in the presence of a number of the neighbors. I commenced by making a semi-lunar incision from a point even with, and just at the right of the umbilicus, passing the knife through the integuments and abdominal muscles down to within two inches of the symphisis pubis, doing which, a large sac or cyst was exposed to view; upon opening the cyst a mass covered with hair was seen in full view. I attempted its removal with my hand, but made a failure; I then seized it with a pair of fanged forceps, and again attempted its removal, but in doing which, one of the fangs broke in consequence of its firm adhesion to the sack posteriorly and inferiorily; on my second trial, I succeeded in the removal of a mass weighing 18 ounces, the upper portion of which, was covered with a thick coat of hair about three inches long. The undecayed portion of the funis was about two inches in length, attached to the centre of the mass, as in cases of newly born infants. At what point in the cyst the cord had been attached, I could not discover. There was a completely formed rectum, on either side of which, was a prominence of considerable size, designed evidently for the nates and hip joints; there were two appendages, evidently intended for an arm and an ear; there were cervical vertebræ, one of the articulations being exposed, evidently caused by the decay and discharge of whatever was attached to it. The mass had evidently retained vitality and continued to grow up to the time of the attack of fever, which was about two years from the child's birth. The sack was cleansed by injecting into it tepid milk and water. The edges of the incision were brought together and retained by adhesive straps, leaving a small opening at the pending portion for the escape of any matter or fluid which might collect in the cyst. The incision healed kindly by first intention, and under the liberal use of bark, wine, and aromatic sulph. acid, the patient rapidly recovered, and was discharged well in about ten days. She is now in her sixteenth year, as hale and healthy a girl as any in Talbot county. I preserved the preparation in alcohol, intending to present it to the Medical College of Augusta, Ga.; but some unprincipled person, not having the fear of God before them, stole it from my office.

